# Central venous catheter-related bloodstream infection and colonization: the impact of insertion site and distribution of multidrug-resistant pathogens

**DOI:** 10.1186/s13756-020-00851-1

**Published:** 2020-12-01

**Authors:** Vassiliki Pitiriga, Petros Kanellopoulos, Ioannis Bakalis, Elsa Kampos, Ioannis Sagris, George Saroglou, Athanasios Tsakris

**Affiliations:** 1 Department of Microbiology, Medical School, National and Kapodistrian University of Athens, 75 Mikras Asias Street, 11527 Athens, Greece; 2 Department of Internal Medicine, Metropolitan Hospital, 9 Ethnarchou Makariou Street, 18547 Athens, Greece

**Keywords:** Catheterization, Central venous catheter, Sepsis, Colonization, Bloodstream infection, Insertion site, Catheter

## Abstract

**Background:**

Placement of central-venous catheters (CVCs) is an essential practice in the management of hospitalized patients, however, insertion at the commonly used sites has often the potential of inducing major complications. Neverthelss, the impact of specific site central line catheter insertion on catheter-associated bloodstream infections (CLABSIs) has not been clarified yet in the literature.

**Objective:**

The aim of the study was to compare CLABSIs and catheter colonization rates among the three catheter insertion sites: subclavian (SC), internal jugular (IJ) and femoral (FEM) in hospitalized patients. Moreover, to analyze the distribution of pathogens and their antimicrobial resistance profiles at these three sites, concurrently.

**Methods:**

We performed a retrospective analysis of data collected prospectively from all catheterized patients at a tertiary care Greek hospital from May 2016 to May 2018. Data was collected on 1414 CVCs and 13,054 CVC-days.

**Results:**

Τhe incidence of CLABSIs among the three sites was as follows: SC:5.1/1000 catheter/days, IJ: 3.73/1000 catheter/days and FEM: 6.93/1000 catheter/days (*p* = 0.37). The incidence of colonization was as follows: SC:13.39/1000 catheter/days; IJ:7.34/ 1000 catheter/days; FEM:22.91/1000 catheter/days (*p* = 0.009). MDROs predominated in both CLABSIs and tip colonizations (59.3 and 61%, respectively) with *Acinetobacter baumanii* being the predominant pathogen (16/59, 27.1% and 44/144, 30.5%, respectively). The incidence of CLABSIs due to multidrug-resistant organisms (MDROs) was as follows: SC:3.83/1000 catheter days; IJ:1.49/1000 catheter days; FEM:5.86/1000 catheter days (*p* = 0.04). The incidence of tip colonization by MDROs among the 3 sites was as follows: SC:8.93/1000 catheter/days; IJ:4.48/1000 catheter/days; FEM:12.79/1000 catheter/days (*p* = 0.06). There was no significant difference in the type of pathogen isolated among site groups for both CLABSIs and tip colonizations.

**Conclusions:**

FEM site of catheter insertion was associated with a higher rate of bloodstream infection and catheters’ colonization compared to IJ and SC sites. Furthermore, this survey highlights the changing trend of the distribution of frequent pathogens and resistance patterns towards MDR Gram-negative pathogens, underscoring the need for consistent monitoring of antimicrobial resistance patterns of these specific infections.

## Introduction

Bloodstream infections (BSI) are a significant cause of morbidity and increased mortality in healthcare facilities; they are also attributed to an increased length of stay and escalating costs [1].Central-line catheter use is a major risk factor for bloodstream infections [2] with more than 250,000 cases of hospital-acquired central line associated bloodstream infections (CLABSIs) within the United States annually [3]. Several factors, such as those related to the patient (i.e. immunodeficiency, renal replacement therapy), central-venous catheter (CVC) use (prolonged catheterization, type of catheter material, and anatomical site of catheter insertion), and healthcare practice (poor barrier methods during catheter insertion and handling) have been shown to increase the risk of CVC infection [4]. Regarding the anatomical site of insertion, the actual risk of infection at the three most commonly used insertion sites: subclavian (SC), internal jugular (IJ) and femoral (FEM), remains controversial. Indeed, although earlier studies supported that FEM site access was associated with a higher risk of infections, previous studies reported no significant differences in the rate of CLABSIs between these three sites, provided that all precaution measures are constantly implemented throughout the hospital settings [5]. Furthermore, while it has been suggested that anatomical insertion site may influence the type of bacteria isolated from catheter tip culture as a cause of CLABSI [6], no studies have compared the three sites in terms of the pathogen distribution and resistance profiles of microorganisms isolated.

The objective of the present study was to compare CLABSIs and catheter tip colonization rates among the three insertion sites in central line catheterized patients as well as to analyze and compare distribution of pathogens and resistance profiles among the three groups.

## Methods

We performed a retrospective analysis of data collected prospectively from consecutive admissions to Metropolitan Hospital, a tertiary care hospital in Athens, covering a 24-month period from May 2016 to May 2018. This observational study was approved by the institutional review board.

### Selection of insertion sites

Triple lumen, non-antibiotic impregnated catheters (Arrow model,total provided by Arrow®/Teleflex®, Wayne, USA), are mainly used in our hospital. They are preferred exclusively for critically ill patients of ICU due to their three infusion channels that facilitate the delivery of several parenteral medications and fluids simultaneously, but also in the others than ICU units. Double lumen catheters (Arrow®/Teleflex®, Wayne, USA), are also used but in a smaller percentage, particularly in patients that do not require complex therapeutic interventions. Catheters were inserted at optimal sites. The choice of the site of insertion was left to the discretion of the physician caring for the patient. The operators were proficient in accessing all three sites. Maximal sterile barrier precautions (large sterile drape; surgical hand antisepsis; and mask, cap, sterile gloves, and gown) were used at catheter insertion according to CDC recommendations. Catheters were immediately removed if no longer needed, or if a catheter-related infection (CRI) was suspected.

### Catheter care

Standardized CVC care was achieved by a highly trained nursing staff proficient in all aspects of CVC care. All insertion sites were maximally visualized for potential dressing contamination. Every couple of days or earlier if clinically required, the nursing staff changed the dressing, cleaned the skin site and the catheter hub with iodine solution, and changed the intravenous accessory tubing. Furthermore, the nursing staff independently enforced sterile insertion technique.

### Data collection

After insertion, catheters were checked using a check-box form containing the patient’s diagnosis, operator’s name, site chosen,date placed and removed, date of intensive care units (ICU) discharge or death, mechanical ventilation, arterial catheters, parenteral nutrition, vasopressor support, and daily clinical assessment (e.g., induration, discharge, erythema, and tenderness) of possible catheter infection. The operator inserting the catheter entered the initial data; nurse personnel entered data the following days while the infection control nurse monitored data collection 3–4 times per week.

We retrospectively collected study data from three different sources where information was completed: 1) ICU database (for demographic and clinical data related to the patient’s admission and clinical course); 2) and 3) Clinical Laboratory and hospital infection control team database (for blood culture and antibiotic susceptibility results).

### Indications for catheter removal

CVCs were removed when a) there was a suspicion of infection, b) when the catheter was no longer required, and c) after 15 days of insertion. Fifteen days’ duration is our institutional policy to avoid catheter infection from excess catheter duration [7]; the duration is based on the incidence of CVC infection from the institution’s infection control program. Occasionally, CVCs remained more than 15 days if the risk of obtaining new venous access was higher than leaving the current CVC in place.

### Culture techniques

All catheters were examined for the presence of pathogens either as a routine after removal or after suspicion of infection. After disinfecting skin around the CVC entry site, the proximal 4–5 cm part of the tip was cut off using sterile scissors. The specimen was placed in a sterile container and transported to the microbiology laboratory within 15 min at room temperature. The intradermal and intravascular portion of the catheter was analyzed by the semiquantitative culture technique described by Maki et al. [8]. According to Maki’s technique, catheter-tip culture is considered positive in the presence of ≥15 colony-forming units (CFU) growth of any organism. Blood cultures were incubated in Becton Dickinson Bactec (BD Bio-sciences, USA) in aerobic and anaerobic broth media. Identification of isolates and antimicrobial resistance patterns were determined by the VITEK®2Automated Compact System (BioMérieux Co., France). E-test (BioMérieux Co., France) was performed as an additional test, in order to confirm the resistance phenotypes reported by the VITEK System, according to the standard laboratory procedures. Multidrug-resistant organisms (MDROs) were defined as **s**pecies of microorganisms that exhibit antimicrobial resistance to at least one antimicrobial drug in three or more antimicrobial categories. This definition concerns both gram-positive and gram-negative bacteria [9].

### Evaluation of CVC infection

The definitions of catheter infection and colonization are based on the Centers for Disease Control bloodstream infection guidelines and the semiquantitative culture technique by Maki et al. [8].

**Catheter associated BSI (*CLABSI)*** was defined as a laboratory confirmed BSI (a positive blood culture with no other apparent source of infection) occurring in the presence of a CVC or within 48 h of CVC removal.

**Catheter colonization:** The presence of ≥15 CFU of a single organism per catheter if not accompanied by a laboratory confirmed BSI of the patient.

*Catheter/days* was defined as the number of CVCs presents among all units’ patients at 08:00 h each morning. When more than one concurrent CVC was present in the patient, they were counted as one CVC.

*Primary bloodstream infection* was confirmed if either criterion 1 or criterion 2 were satisfied:

1. Patient had a recognized pathogen cultured from one or more blood cultures
2. If the organism cultured was considered a common skin contaminant (Diphtheroids, Bacillus species, coagulase-negative Staphylococcus or Micrococcus) and patient had at least one of the following signs or symptoms: fever (38 °C) or hypotension and positive laboratory results were not related to an infection at another site, and at least one of the following:
  Organism cultured from two or more blood cultures drawn on separate occasions.
  Organism cultured from at least one blood culture from a patient with an intravascular line, and the physician instituted appropriate antimicrobial therapy.

The clinical status of the patients was assessed daily by on-site attendings. The insertion sites were examined routinely as part of the clinical assessment. Potential CVC infection was considered any time an infection occurred.

### Statistical analysis

Characteristics of patients and catheters were described as count (percent) or mean value (+/− standard deviation) for qualitative and quantitative variables, respectively, and were compared between the three catheters groups using Chi-square or T-test, as appropriate. Non-normally distributed quantitative variables were expressed as median (interquartile range). The one-way ANOVA test was used to determine the existence of statistical significant difference in CLABSIs, colonization rates, catheter duration at the three insertion sites. A *p*-value of ≤0,05 was considered as statistically significant.

## Results

A total of 13,054 catheter days and 1414 catheters were analyzed. Of them, 249 (3136 catheter days) were of SC site, 945 (8041 catheter days) of IJ site and 220 (1877 catheter days) of FEM site. The mean duration of catheterization was 12.59 ± 8.32 [95% CI 11.6 to13.6] days for SC sites, 8.51 ± 6.45 (95% CI 7.59 to 8.41) days for IJ sites and 8.53 ± 8.13(95% CI 6.93 to 9.07) days for FEM sites (*p* = 0.03, ANOVA). The overall incidence of CLABSIs was 5.32/1000 catheter days, and the overall catheter colonization rate was 12.48/1000 catheter days.

The distribution of CLABSIs events among hospital units was as follows: 25/59 (43%) in ICU; 20/59 (35%) in internal medicine department; 14/59 (19%) in other units. The distribution of catheter colonizations among hospital units was 62/144 (37%) for ICU, 42/144 (29%) for internal medicine department, 40/144 (12%) for others. The total patients’ demographic characteristics in cases of CLABSIs and tip colonization are presented in Table 1. The 3 groups of patients with different catheter insertion sites had equally distributed demographic characteristics and severity of illness (as defined by APACHI score; data not shown).

**Table 1:** Study populations’ demographic and clinical characteristics

VARIABLE	No of patients	
	CLABSI (***n*** = 59) N (%)	Colonization (***n*** = 144) N (%)
Age, mean +/− SD, (years)	55.08 +/− 19.8	53.9 +/− 17.9
Gender (M/F)	41/18	100/44
Obesity	19 (32.2)	84 (58.3)
Diabetes mellitus	9 (15.2)	67 (46.5)
Pulmonary disease	16 (27.1)	48 (33.3)
Hypertension	11 (18.6)	75 (52)
Renal disease	17 (28.8)	15 (10.4)
Oncologic disease	16 (27.1)	36 (25)
Immune deficiency/suppression	17 (28.8)	33 (22.9)
Admission category		
Medical	43 (72.8)	87 (60.4)
Surgery	16 (27.1)	57 (39.6)
Mechanical ventilation	37 (62.7)	105 (72.9)
Unit of cath insertion (ICU/other)	38/21	97/48
Cardiovascular disease	17 (28.8)	49 (34)
Neurological disease	38 (64.4)	29 (20.1)
Gastroenterological disease	18 (30.5)	27 (18.7)
Hospital death	19 (32.2)	42 (29.1)
Sepsis	11 (18.6)	29 (20.1)
APACHE score	12.8+/− 8.2	13.2 +/− 7.4
Length of catheter stay (mean +/− SD)	16.19+/−10.7	14.05+/− 9.7

*No* number of catheters

The median length of stay prior to central line insertion was 16 days (Interquartile range = 55) in CLABSIs by MDROs and 25 days (Interquartile range = 57) in CLABSIs by other pathogens (*p* = 0.13). In tip colonizations, the median length of stay prior to central line insertion was 10 days (Interquartile range = 36.75) in MDROs cases and 14 days (Interquartile range = 64.25) in cases where other pathogens were involved (*p* = 0.40). The mean duration of catheterization until the onset of infection was 16.1 ± 10.7 days in CLABSIs cases and 14. ± 9.7 days in colonization cases. In 47.45% (28/59) of CLABSIs cases and 34.7% (50/144) of colonization cases the duration of catheterization was ≥15 days.

A higher incidence of CLABSIs was observed in catheters of FEM site as compared to the other 2 sites, although not in a statistically significant level. More specifically, the incidence of CLABSIs among the 3 sites was as follows: SC:5.1 infections per 1000 catheter days, IJ:3.73 infections per 1000 catheter days, and FEM:6.93 infections per 1000 catheter days (*p* = 0.37; ANOVA). Regarding catheter colonization, a higher incidence of infection was also observed in FEM catheters compared to the other sites that was established in a statistically significant level. More specifically, the incidence of colonization was as follows: SC:13.39 per 1000 catheter days; IJ:7.34 per 1000 catheter days; and FEM:22.91 per 1000 catheter days (*p* = 0,009; ANOVA) (Table 2). Similarly, the incidence of CLABSIs and tips colonization from MDROs was higher in FEM site as compared to the others. More specifically, the incidence of CLABSIs by MDROs was as follows: SC: 3.83 infections per 1000 catheter days; IJ:1.49 per 1000 catheter days; FEM:5.86 per 1000 catheter days (*p* = 0.04; ANOVA) (Table 2). The incidence of colonization by MDROs among the three sites was as follows: SC:8.93 per 1000 catheter days; IJ:4.48 per 1000 catheter days; FEM:12.79 per 1000 catheter days (*p* = 0.06; ANOVA) (Table 2). No significant difference in the proportion of MDROs associated with colonization (88/144 = 61%) vs infection (35/59 = 59.3%) was observed (*p* = 0.91, Pearson chi-square).

**Table 2:** CLABSI and tip colonization rates among the 3 sites of catheterization

	SC	IJ	FEM	***P–***value
**TOTAL ISOLATES**				
**CLABSI (No)**	16	30	13	X^2^ = 6.53 *p* = 0.03
Per 1.000 catheter/days	5.1	3.73	6.93	ANOVA F = 1.06 *p* = 0.37
**Tip colonization (No)**	42	59	43	X^2^ = 38.74, *p* = 0.000
Per 1.000 catheter/days	13.39	7.34	22.91	ANOVA F = 6.23 *p* = 0.009
**MDROs**				
**CLABSI (No)**	12	12	11	X^2^ = 16.14, *p* = 0.000
Per 1000 catheter/days	3.83	1.49	5.86	ANOVA F = 3.66 *p* = 0.04
**Tip colonization (No)**	28	36	24	X^2^ = 24.59, *p* = 0.000
Per 1000 catheter/days	8.93	4.48	12.79	ANOVA F = 3.05 *p* = 0.06

*p*≤ 0.05 significant

The duration (in days) of catheterization until the onset of infection was not statistically different among the 3 insertion sites with regards to CLABSIs in total (*p* = 0.77, ANOVA) and CLABSIs from MDROs (*p* = 0.83, ANOVA). In tip colonization longer catheter duration was observed in SC insertion site as compared to the other two (*p* = 0.002 ANOVA) (Table 3). Similarly, no differences in age were observed between the 3 sites with regards to CLABSIs from MDROs (*p* = 0.14 ANOVA), while patients with SC catheters were significantly younger than those with IJ or FEM catheters in tip colonizations and CLABSIs in total (*p* = 0.002 and *p* = 0.001 respectively ANOVA) (Table 3). No difference in tip colonization rates of the 3 sites was noted between males and females, while CLABSIs in IJ site were more frequent in females compared to males (*p* < 0,004 Pearson chi-square) (Table 3).

**Table 3:** Duration of catheter stay, age of patients and gender proportions among the 3 sites of catheterization

	N	Mean	Std. Deviation	95% confidence interval		***P***-value
**CLABSIs**						
**Duration**				Lower Bound	Upper Bound	
FEM	13	15.23	16.13	5.48	24.98	0,77
IJ	30	15.73	8.31	12.63	18.84	
SC	16	17.81	10.1	12.43	23.19	
**Age**						
FEM	13	58.23	21.71	45.11	71.35	**0.001**
IJ	30	61.73	16.75	55.48	67.99	
SC	16	40.06	16.24	31.41	48.72	
**TIP COLONIZATION**						
**Duration**						
FEM	43	11.42	11.1	8.00	14.84	**0.002**
IJ	59	12.85	7.84	10.80	14,89	
SC	42	18.43	9.56	15.45	21.41	
**Age**						
FEM	43	56.74	19.70	50.68	62.81	**0.002**
IJ	59	57.61	16.45	53.32	61.9	
SC	42	45.79	15.85	40.85	50.73	
**MDROs in CLABSI**						
**Duration**						
FEM	11	14.4	15.43	3.36	25.44	0.83
IJ	12	13.67	7.59	8.84	18.49	
SC	12	16.56	8.6	9.94	23.17	
**Age**						
FEM	11	57.7	21.84	42.07	73.33	0.14
IJ	12	64.08	16.53	53.57	74.59	
SC	12	47.44	16.4	34.84	60.05	

Gender			SC *n* (%)	IJ *n* (%)	FEM *n* (%)	*P*-value
**CLABSI**						
Male			11 (26.8)	15 (36.6)	15 (36.6)	**0.004**
Female			2 (11.1)	15 (83.3)	1 (5.6)	
**Tip Colonization**						
Male			31 (31.3)	36 (36.4)	32 (32.2)	0.23
Female			12 (26.7)	23 (51.1)	10 (22.2)	

*n* number; *p*≤ 0.05 significant

A total of 18 different microorganisms were responsible for the 144 CVC tip colonization’s while in CLABSIs a total of 12 different microorganisms were isolated. Microorganisms involved in the 3 types of catheters’ infection/colonization are presented in Table 4. There was no significant difference in the type of pathogen isolated between the site groups for both CLABSIs and tip colonizations. MDROs included *Acinetobacter baumanii*, *Pseudomonas aeruginosa* and *Klebsiella pneumoniae* and two isolates of *Proteus mirabilis.* In CLABSIs the types of pathogens were as follows: In FEM: 10 (76.9%) Gram- negatives, 2 (15.4%) Gram-positives and one (7.7%) yeast; in IJ: 16 (53.3%) Gram-negatives, 10 (33.3%) Gram-positives and 4 (13.3%) yeasts; in SC; 12 (75%) Gram-negatives, 3 (18.7%) Gram-positives and one (6.2%) yeast. In tips colonization the types of pathogens were as follows: In FEM: 32 (74.4%) Gram-negatives, 5 (11.6%) Gram-positives and 6 (13.9%) yeasts; in IJ: 48 (81.3%) Gram-negatives, 5 (8.4%) Gram-positives, 5 (8.4%) yeasts; in SC: 31 (73.8%) Gram-negatives, 5 (11.9%) Gram-positives and 6 (14.3%) yeasts (Table 4).

**Table 4:** Pathogen distribution among tip colonizations and CLABSIs

	CLABSIs *n* (%)			TIP COLONIZATION *n* (%)		
	SC	IJ	FEM	SC	IJ	FEM
**Gram-negative bacteria**						
MDR *K. pneumoniae*	5/31.2	2/6.6	6/46.1	9/21.4	10/16.9	8/18.6
MDR *A. baumannii*	2/12.5	10/33.3	4/30.7	13/30.9	19/32.2	12/27.9
MDR *P. aeruginosa*	3/18.7	–	–	5/11.9	4/6.7	3/6.9
MDR *P. mirabilis*	–	–	–	–	1/1.6	1/2.3
Non-MDR *K. pneumoniae*	*–*	–	–	1/2.3	1/1.6	1/2.3
Non-MDR *P. aeruginosa*	–	1/3.3	–	1/2.3	5/8.4	1/2.3
*E. coli*	–	–	–	1/2.3	4/6.7	4/9.3
**Gram-positive bacteria**						
*Coagulase-negative staphylococci*	1/6.2	8/26.6	1/7.6	2/4.7	2/3.3	4/9.3
Methicillin-resistant *S. aureus* (MRSA)	2/12.5	–	1/7.6	1/2.3	2/3.3	–
*Enterococcus spp.*	–	2/6.6	–	1/2.3	1/1.6	–
**Yeasts**						
*Candida* spp.	1/6.2	4/13.3	1/7.6	6/14.2	5/8.4	6/13.9
**Other bacteria**	2/12.5	3/10	–	2/4.7	4/6.7	2/4.6

*n* number

## Discussion

The present study has shown that CLABSIs and catheter tip colonization rates are differing among the three commonly used CVC insertion sites. Indeed, a significantly increased rate of colonization, as well as a trend towards increased rate of CLABSIs in the FEM site were observed in comparison to the other two sites. Notably, although most CVCs were inserted via the IJ vein (66.8%), yet their contribution to CLABSIs was the lowest.

In the literature conflicting data exist regarding the risk of acquiring infection for the three sites of catheterization, with studies supporting higher risk at the FEM site and lower at the SC [10], while others reporting no difference [11, 12]. The latter was found even in studies conducted in immunocompetent patients when optimal insertion sites are selected, experienced operators insert the catheters, strict sterile technique is present, and trained intensive care unit nursing staff perform catheter care. Furthermore, in a multicenter study where patients were catheterized through FEM or SC site, CVC placement in the FEM area was associated with a substantially greater risk of CLABSI compared to the SC insertion [13]. In a recent survey, insertion into the FEM and IJ vein was independently associated with an increasing risk of subsequent CLABSI [14], while in another, the SC site was associated with the lowest risk of infection when compared to the other two sites [15]. Since it is well known that the risk of catheter infections is in part related to the density of local skin flora [16], FEM site carries obvious sources of contamination from groin secretions. Therefore, many of the clinical practice guidelines recommend that the FEM site should be avoided due to the perceived higher risk of CLABSIs associated with this site [17]. However, the preferred site for placement of a CVC is complex and based on the skill and expertise of the operator, the availability and expertise of ultrasound-guided placement, the risk of bleeding and other complications (pneumothorax), as well as the urgency of placement. In emergent and high-risk situations, the FEM route is often chosen due to the ease and perceived lower insertion risk of this site [18].

In our study, Gram-negative pathogens and especially MDROs predominated in CLABSIs as well in catheter colonization, followed by Gram-positive and yeast infections, with MDR *A. baumanii* being the predominant pathogen (16/59, 27.1% and 44/144, 30.5%, respectively). The results are also suggesting that the FEM site carries a greater risk of infection than either the IJ or SC sites for organisms other than coagulase-negative staphylococci. This may have some implications for treatment of suspected CLABSI arising from the FEM site where organisms of significantly greater virulence may be responsible.

According to the international CLABSI pathogen distribution patterns published in several previous studies, the microorganisms causing CRIs usually belong to the normal resident flora of the skin at the insertion site, mainly Gram-positives such as *Staphylococcus aureus*, *Staphylococcus epidermidis, Streptococcus* spp., *Enteroccoccus* spp.*, Corynebacterium* spp., and fungi such as *Candida* spp. [19–21]. However, recently published data have revealed that CVC infections by Gram-negatives either predominated in terms of pathogen distribution or showed increasing trends [22–24]. In our study the pathogen distribution of catheter infections follows the recently reported Greek ICU profile of central line colonization and infection pathogens, where the incidence of *A. baumanii* is high, possibly due to the local predominance of Gram-negative microorganisms [25]. The emergence of MDR bacteria has created a new burden on medical care in Greek hospitals, particularly for patients admitted to ICU [25]. This fact, might have contributed to the domination of these pathogens in the microbial profile of catheter infection in the current study. Indeed, in our institute, the rates of the 3 most commonly isolated MDR Gram-negatives (61% *A. baumanii*, 25% *K. pneumoniae,* 14% *P. aeruginosa*) for the same time period was 21.6% among hospitalized patients for all clinical specimens, 63.3% of which were isolated from ICU (data not shown). Moreover, 43% of CLABSIs by MDROs and 59% of colonizations by MDROs were recovered from ICU patients, whereas all patients from other units had a history of prior admission to an ICU before the isolation of MDROs. In addition, it should be also noted that in a significant proportion of CRIs (47.45% of CLABSIs and 34.7% of colonization cases) the duration of catheterization was ≥15 days. This is a highly contributing factor for catheter infection and colonization that should be acknowledged. On the contrary, as concerns the length of hospital stay prior to central line insertion, no evidence from our analysis has proven that it consists a causative agent for the colonization by MDROs. In terms of the differences in catheterization duration among the three sites, we have made an attempt to search for potential reasons to justify the longer SC catheter duration compared to the other two sites, however, based on the existing data, we have not reached any definite conclusion.

## Conclusion

The findings of the present study support that IJ and SC insertion sites are safer to use compared to the FEM site as concerns both CLABSIs and catheter colonizations. Moreover, a significant shift in the epidemiology of CRIs towards a predominance of Gram-negative pathogens and especially MDROs was observed. Since MDR Gram-negative infections are linked to significant mortality rates, empirical treatment should take their increasing prevalence into consideration.

## Data Availability

All data generated or analysed during this study are included in this published article.

## Abbreviations

CLABSI: Catheter associated blood stream infection
CRI: Catheter-related infection
CVC: Central-venous catheters
FEM: Femoral
IJ: Internal jugular
MDRO: Multidrug-resistant organism
SC: Subclavian
